# Molecular Pathological Markers Correlated With the Recurrence Patterns of Glioma

**DOI:** 10.3389/fonc.2020.565045

**Published:** 2021-01-08

**Authors:** Shunnan Ge, Yingwu Shi, Gang Zhu, Songlun Li, Yaning Cai, Peigang Ji, Jinghui Liu, Wei Guo, Li Gong, Miao Lou, Fuqiang Feng, Yuan Wang, Yulong Zhai, Yan Qu, Liang Wang

**Affiliations:** ^1^ Department of Neurosurgery, Tangdu Hospital, Fourth Military Medical University, Xi’an, China; ^2^ Medical Department of Tangdu Hospital, Fourth Military Medical University, Xi’an, China; ^3^ Department of Pathology, Tangdu Hospital, Fourth Military Medical University, Xi’an, China; ^4^ Department of Neurosurgery, The Second Hospital of Shanxi Medical University, Taiyuan, China

**Keywords:** glioma, local recurrence, paraventricular recurrence, molecular markers, O6-Methylguanine DNA methyltransferase

## Abstract

**Purpose:**

Glioma is one of the most common tumors of the central nervous system, and many patients suffer from recurrence even after standard comprehensive treatment. However, little is known about the molecular markers that predict the recurrence patterns of glioma. This study aimed to demonstrate the correlations between molecular markers and glioma recurrence patterns, which included local/nonlocal recurrence and paraventricular/nonparaventricular recurrence.

**Methods:**

Immunohistochemical techniques were used to assess the molecular markers of 88 glioma tissues following surgical resection. The recurrence patterns were divided into local recurrence, marginal recurrence, distant recurrence, multirecurrence, and subarachniod recurrence, with the last four recurrence patterns being collectively called nonlocal recurrence. According to whether the recurrence invaded ventricles, the nonlocal recurrence patterns were divided into paraventricular and nonparaventricular recurrence. Then, we compared the different recurrence patterns and their clinical characteristics, focusing on the expression of molecular markers.

**Results:**

More patients in the nonlocal recurrence group received combined radiotherapy and chemotherapy than patients in the local recurrence group (p=0.019). Sex, age, extent of surgery, time to recurrence, tumor location, size, and WHO grade were not different in the defined groups (P>0.05). Recurrent tumor volume and WHO grade were significantly different between the paraventricular and nonparaventricular recurrence groups (p=0.046 and 0.033). The expression of Ki-67, P53, and PCNA in the nonlocal recurrence group was significantly higher than that in the local recurrence group (p=0.015, 0.009, and 0.037), while the expression of S-100 in the nonlocal recurrence group was significantly lower than that in the local recurrence group (p=0.015). Cox regression indicated hazard ratio (HR) for high expression level of PCNA associated with non-local recurrence was 3.43 (95% CI, 1.15, 10.24), and HR for high expression level of MGMT associated with paraventricular recurrence was 2.64 (95% CI, 1.15,6.08).

**Conclusions:**

Ki-67, P53, PCNA, and MGMT might be important clinical markers for nonlocal recurrence and paraventricular recurrence.

## Introduction

As the most common type of primary intracranial tumor, gliomas frequently lead to severe neurological deficits, with some subtypes showing very pessimistic outcomes, and treatment for gliomas is still a great challenge (1). For low-grade gliomas (LGGs), such as WHO grade II infiltrating astrocytoma and oligodendroglia, maximal safe resection is still the key factor for a better prognosis, and radiotherapy and chemotherapy may be necessary for some high-risk cases (2). For high-grade gliomas (HGGs), such as WHO grade III anaplastic gliomas and glioblastoma (GBM), surgical treatment combined radiotherapy and chemotherapy is the standard strategy for most patients (3).

Though great effort has been made to optimize comprehensive therapy for gliomas, the prolonged survival of patients is still limited. In particular, the standard Stupp strategy only results in an improvement in the median survival from 12.1 months to 14.6 months and the median progression-free survival (PFS) from 5.0 months to 6.9 months for GBM (4). Among the factors leading to the short survival of glioma patients, a high incidence of recurrence is the greatest challenge (5); thus, analyzing the patterns of recurrence and its relevant factors may be of great significance for the prognosis of patients. Previous studies have focused on the patterns of recurrence for GBM after surgical resection, temozolomide (TMZ)-based chemotherapy, and radiotherapy with different protocols, as well as bevacizumab treatment, all revealing central recurrence to be the main pattern (6, 7). According to Alba A. Brandes, MGMT promoter methylation status was found to be correlated with the site of recurrence for GBM (8). For patients with LGGs undergoing radiotherapy, recurrence uniformly occurred within the treatment volume, suggesting that dose escalation for radiotherapy within conformal fields could improve the treatment outcome (9).

The present study reported a consecutive glioma series with posttreatment recurrence, and the possible factors impacting the patterns of recurrence, including treatment strategy and histopathology, were analyzed. In addition, the correlations between several molecules crucial to the biochemical behavior of tumors and the patterns of recurrence were investigated.

## Materials and Methods

### Patient Population

This retrospective study was conducted on a consecutive glioma series, enrolling 145 patients who underwent surgical treatment in our institution from January 1, 2016, to October 15, 2019. All selected patients were histopathologically diagnosed with diffuse astrocytoma, oligodendroglioma, anaplastic astrocytoma, oligodendroglioma and GBM. A total of 45 patients who were lost to follow-up or had no follow-up magnetic resonance imaging (MRI) scans available were excluded, and 12 patients did not experience recurrence during the follow-up period. Ultimately, 88 patients were included in this study. This retrospective study strictly complied with the Declaration of Helsinki (Sixth Revision, 2008). All data were collected retrospectively and in accordance with the ethical policies of our institution.

### Surgery and Postoperative Treatment

Demographic information, including age, sex, presenting symptoms, and preoperative Karnofsky Performance Status (KPS) score, was collected, and preoperative T2/flair or T1-weighted contrast-enhanced imaging was reviewed to determine tumor location and volume. Then, all patients underwent maximal safe tumor resection. All patients underwent postoperative MRI scans within 48 h after each surgery, and the extent of resection was made by a side-by-side comparison of the pre- and post- operative images, following the criteria as follows: Gross total resection (GTR) was defined as the absence of hyperintense tissue on T2-weighted/fluid-attenuated inversion recovery (FLAIR) sequences (LGGs) or the absence of contrast-enhancing tissue on T1-weighted sequences (HGGs); subtotal resection (STR) was classified as anything other than GTR (10).

After the final histopathological diagnosis was established (according to WHO criteria), the patients underwent observation and only radiotherapy, only chemotherapy, or combined radiotherapy and chemotherapy (containing Stupp treatment) according to the National Comprehensive Cancer Network (NCCN) guidelines (11). Temozolomide (TMZ) protocol was applied in chemotherapy for cases of all pathological types.

### Molecular Marker Status Assessment

The postoperative tumor tissues were promptly sent to the Pathology Department for WHO grading. The molecular pathological results of the patients were determined by immunohistochemistry. For the cases after the year 2017, 1p 19q co-deletions was examined for the diagnosis of oligodenrogliomas. The molecular markers are shown in **Table 1**.

**Table 1:** Molecular markers in this study.

Abbreviation	Full names
GFAP	Glial fibrillary acidic protein
Olig-2	Oligodendrocyte precursor
Syn	Synaptophysin
NSE	Neuron-specific enolase of Enzyme
Pgp170	P-glycoprotein 170
Vimentin	–
MGMT	O6-Methylguanine DNA methyltransferase
MMP-9	Matrix metalloproteinase-9
IDH-1	Isocitrate dehydrogenase-1
EGFR	Epidermal growth factor receptor
GST-π	Glutathione S-transferase-π
Topo-II	Topoisomerase II
Ki-67	Antigen KI67
PCNA	Proliferating cell nuclear antigen
S-100	Soluble protein-100
P53	–

Brown cell nucleus and/or cytoplasm and/or cytomembrane staining of the target molecular protein is positive staining. The expression of Ki-67 was a continuous variable according to the ratio of positive cell numbers. According to the ratio of positive staining and staining intensity, the remaining immunohistochemistry results were divided into five classes: negative (-, no cell staining), weak positive (+-, yellow staining or positive cell number<10%), positive (+, light-brown staining or positive cell number>10%), and strong positive (++, deep-brown staining or positive cell number>25%). Not the number of positive cells was simply compared between the different recurrence types, the expressive intensity identified the above-mentioned classification for immunohistochemistry results was deliberately compared.

### Follow-Up

HGG patients underwent brain MRI 2-6 weeks after radiotherapy, then every 2–4 months for 3 years and every 6 months indefinitely. LGG patients underwent brain MRI every 3–6 months for 5 years and then at least annually, or according to clinical symptoms: if the patients complained headache, vomiting/nausea, epilepsy, and focal neurological deficits, the immediate image and clinical follow-up should be conducted. The initial and cumulative recurrences of HGG were confirmed referring to the Response Assessment in Neuro-Oncology (RANO) criteria: an increase in 25% of the product of perpendicular diameters of enhancing lesions, a significant increase in the T2/FLAIR nonenhancing component, or the appearance of new lesions. For LGGs, recurrence was defined as an unequivocal increase (25%) in tumor size on follow-up T2/FLAIR MRI or the appearance of new lesions. For LGGs, the appearance of contrast enhancement on an initially nonenhancing lesion in combination with rapid tumor growth indicated possible malignant transformation in addition to recurrence, which should be confirmed by histopathology. Patients with suspicious pseudoprogression were observed without changing adjuvant chemotherapy. If the lesions were stable or resolved during follow-up, these patients were considered to have pseudoprogression. Positron emission tomography imaging or surgical procedures were performed for differential diagnosis as necessary.

### Recurrence Patterns Analysis

**Figure 1:**
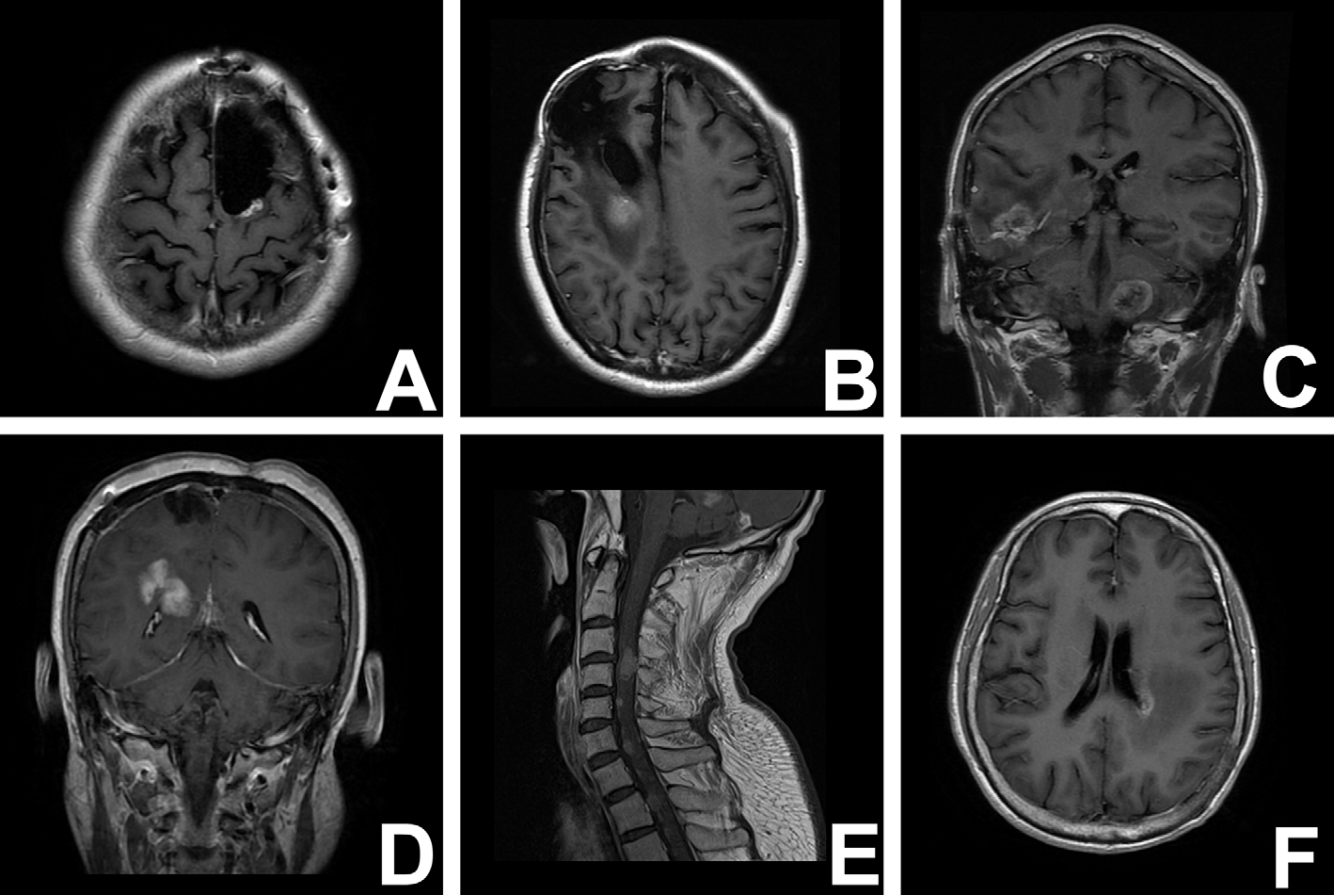
T1-weighred magnetic resonance images illustrate the different recurrent patterns of glioma. **(A)** local recurrence; **(B)** marginal recurrence; **(C)** multirecurrence; **(D)** distant recurrence; **(E)** subarachniod recurrence; **(F)** paraventricular recurrence.

The patterns of recurrence were considered according to the distance of the recurrent tumors from the resection cavity. As described by Yoshiyuki Konishi (12), recurrences were defined as “regional” if tumor recurrence was located in the wall of the resection cavity, “marginal” if tumor recurrence was located within 20 mm from the margin of the resection cavity, “distant” if tumor recurrence was located more than 20 mm from the margin of the resection cavity, “multiple” if tumor recurrence was located in various brain areas, and “subarachnoid” if tumor recurrence was subarachnoid dissemination (12). Recurrence adjacent to (<10 mm) the wall of the lateral ventricle was defined as a unique subgroup in the nonlocal recurrence conditions (**Figure 1**).

### Data Analysis

Statistical analysis was performed using the SPSS Version 21.0 statistical software package (SPSS Inc., Chicago, IL). According to the abovementioned criteria, all patients were divided into the following groups: the local recurrence group and nonlocal recurrence group, which was further divided into the paraventricular recurrence and nonparaventricular recurrence groups. The demographic and clinical data were compared between the groups using the Mann-Whitney U test for continuous variables and the chi-square test for categorical/ordinal variables, while 25% of cells with expected values <5 was subjected to the F exact test. Especially, for the expression level of different molecular markers, except the Ki 67 which was continuous variable, all the other ones were ordinal variables. Univariate Cox regression analysis was performed for identification of independent predictive factors, with development of different patterns of recurrence as the outcome measures. and a multivariate analysis for the factors below a predefined p value <0.1 was further conducted. A two-sided p value of <0.05 was considered statistically significant.

## Results

### Recurrence Conditions

The incidences of various recurrence patterns are presented in **Tables 2** and **3**. Eighty-eight patients were admitted for further analysis, and 43 patients (48.86%) had local tumor recurrence. In addition, marginal recurrence occurred in 21 patients (23.86%), distant recurrence in five patients (5.81%), multirecurrence in 16 patients (18.18%), and subarachnoid recurrence in three patients (3.41%). These four recurrence patterns (45 patients, 51.14%) were included in the nonlocal recurrence group. Additionally, nonlocal recurrence was divided into paraventricular recurrence and nonparaventricular recurrence. The paraventricular recurrence group (32 patients, 71.11%) contained 15 patients (33.34%) with marginal recurrence, two patients (4.45%) with distant recurrence, 14 patients (15.91%) with multirecurrence, and one patient (1.18%) with subarachnoid recurrence. The nonparaventricular recurrence group (13 patients, 28.89%) contained 6 patients (13.33%) with marginal recurrence, three patients (6.67%) with distant recurrence, six patients (2.27%) with multirecurrence, and two patients (2.27%) with subarachnoid recurrence.

**Table 2:** Incidence of local/nonlocal recurrence patterns.

Pattern of recurrence		Cases
Local recurrence		43
Non-local recurrence	Marginal	21
	Distant recurrence	5
	Multirecurrence	16
	Subarachniod recurrence	3
Total		88

**Table 3:** Incidence of paraventricular/non-paraventricular recurrence patterns.

	Paraventricular recurrence	Non-paraventricular recurrence
Marginal recurrence	15	6
Distant recurrence	2	3
Multirecurrence	14	2
Subarachniod recurrence	1#	2
Total	32	13

#also is multi recurrence.

For WHO grade and IDH-1 expression status significantly influenced the prognosis of the glioma patients, (1) the frequency of different recurrence patterns between WHO II/III and WHO IV patients was compared, which showed no significant difference (χ^2^ = 8.221, p=0.065, shown in **Table 4**); (2) the frequency of different recurrence patterns between IDH (+) and IDH(-) patients was compared, which showed no significant difference either(χ^2^ = 4.557, p=0.317, table not shown).

**Table 4:** Comparison of frequency for different recurrence patterns between WHO II/III and WHO IV patients.

	WHO II/III	WHO IV	χ^2^-value	p-value
Local recurrence	11	32	8.221	0.065
Marginal recurrence	3	18		
Distant recurrence	4	1		
Multirecurrence	5	11		
Subarachniod recurrence	1	2		
Total	24	64		

### Baseline Condition

The patient characteristics are summarized in **Table 5**. The number of patients with local recurrence aged >40 years was less than that of patients with nonlocal recurrence (48.84% vs. 71.11%, χ^2^ = 4.554, p=0.033), but the median age between the define groups was not significantly different. Fewer patients with local recurrence received radiotherapy combined with chemotherapy (34.88% vs. 55.56%), which is opposite to an increase in chemotherapy only or radiotherapy only (41.86% vs. 20.00%), and no postoperative treatment was coincident in the two groups (23.26% vs. 24.44%). The number of local recurrence patients receiving radiotherapy combined chemotherapy was significantly less than that of patients receiving chemotherapy only or radiotherapy only (χ^2^ = 5.486, p=0.019). The number of patients with IDH-1 positive and negative expression was similar between the local recurrence group and the nonlocal recurrence group as well (χ^2^ = 0.418, p=0.708) There was no correlation between local tumor recurrence and sex. Although complete resection was achieved in less patients in the local recurrence group than in the nonlocal recurrence group, the extent of surgery did not differ significantly between the two groups (44.19% vs. 60.00%, χ^2^ = 2.204, p=0.138). The number of patients with WHO grade IV was similar between the local recurrence group and the nonlocal recurrence group (72.09% vs. 71.11%, χ^2^ = 0.010, p=0.919). There was no difference in tumor size or location between the defined groups (p=0.729 and 0.362, respectively). The median time to recurrence was not significantly different between the local recurrence and nonlocal recurrence groups (10 (6-19) vs. 10 (7.5-15), U=1.151, p=0.250). The similar comparisons were further done separately in WHO II/III or WHO IV patients, we can only detected that portion of patients with local recurrence aged >40 years showed significantly difference between local and nonlocal recurrence for WHO II/III patients(χ2 = 6.254 p=0.012, **Supplementary Tables 1** and **2**)

**Table 5:** Comparison of clinical factors in patents with different recurrence patterns.

	Local recurrence (n=43)	Non-local recurrence (n=45)	χ^2^- or U- value	p-value	Paraventricular recurrence (n=32)	Non-paraventricular recurrence (n=13)	χ^2^- or U- value	p-value
Sex								
male	20	23	0.289	0.591	15	8	0.795	0.372
female	23	21			17	5		
Age								
median	42(31,52)	47 (37,54)	1.666	0.096	49.5(40,54)	43(33.5,49.5)	1.354	0.176
<40	22	13	4.554	0.033*	7	6	2.653	0.103
>40	21	32			25	7		
Post-operative treatment			5.505	0.064			1.484	0.233
Observe	10	11			7	4		
Chemotherapy only or radiotherapy only	18	9	5.148	0.019*	8	1		
Combine radiotherapy and chemotherapy	15	25			17	8		
Extent of surgery								
Gross-total resection	24	18	2.204	0.138	12	6	0.288	0.591
Subtotal resection	19	27			20	7		
Recurrent tumor volume(mm^3^)	28.51(9.34,79.20)	37.21(8.43,103.36)	0.346	0.729	68.92(11.14,113.00)	9.79(4.63,56.66)	2.135	0.033*
Recurrent tumor location			2.349	0.672			2.421	0.659
frontal	19	24			18	6		
temporal	11	10			7	3		
parietal	3	6			6	0		
occpital	1	3			2	1		
other	9	17			12	5		
WHO grades								
II/III	12	13	0.010	0.919	6	7	3.966	0.046*
IV	31	32			26	6		
IDH-1 status #								
IDH-1(+)	24	26	0.148	0.701	21	5	3.779	0.052
IDH-1 (-)	12	12			6	6		
Time to recurrence (months)	9.5(6,19.75)	8(5,12)	1.151	0.250	7.5(5,11)	9(5,29)	0.906	0.365
Ki-67	10(5,30)	20(10,40)	2.436	0.015*	20(10,40)	12(10,70)	0.240	0.810

*P < 0.05 # Some cases lack the IDH-1 examination.

Also as shown in **Table 5**, there were more WHO IV patients in the paraventricular recurrence group (81.25% vs. 46.15%, χ^2^ = 3.966, p=0.046). The tumor size in the paraventricular recurrence group was greater than that in the nonparaventricular recurrence group (68.92(11.14–113.00) vs. 9.79(4.63–56.66), U=2.135, p=0.033). Sex, age, and tumor location and IDH-1 expression status were not significantly different between the paraventricular recurrence group and the nonparaventricular recurrence group. More patients in the paraventricular recurrence group received radiotherapy combined with chemotherapy (40.63% vs. 30.77%) or chemotherapy only or radiotherapy only (37.50% vs. 38.46%) and were observed (21.88% vs. 30.77%). There were no significant differences in postoperative treatment between the groups (χ^2^ = 1.484, p=0.233). The extent of surgery and time to recurrence were not correlated with the groups. The similar comparisons were further done separately in WHO II/III or WHO IV patients, we can only detected that the significantly different recurrent tumor volumn between paraventricular and non paraventricular recurrence for WHO II/III patients(94.63 (34.14–143.00) vs. 12.04(1.35–70.00), U=2, p=0.046, **Supplementary Table 1-2**)

### The Expression of Molecular Markers by Immunohistochemistry

**Figure 2:**
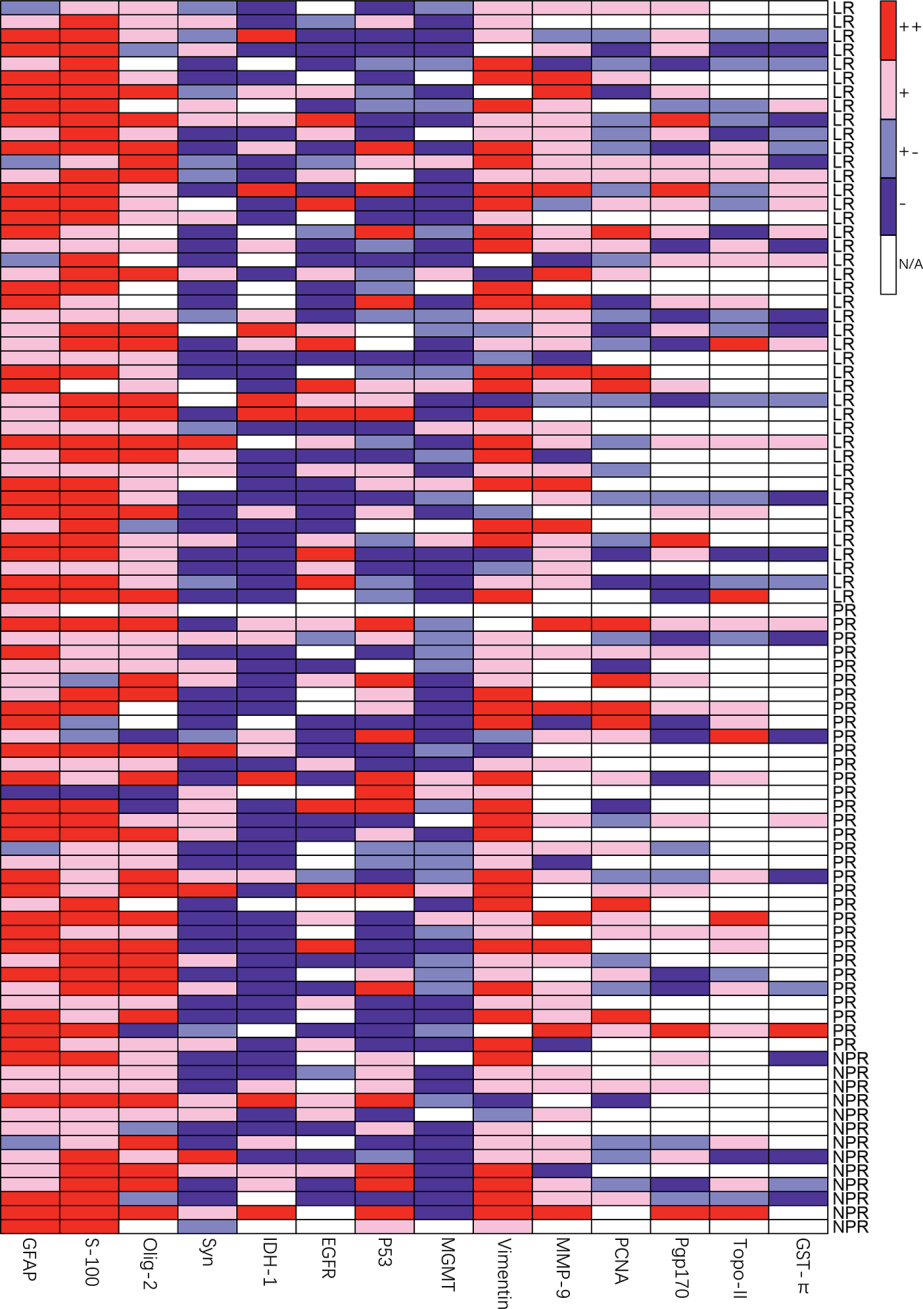
Heatmap of relative molecular markers expression of different groups, LR, local recurrence; PR, paraventricular recurrence; NPR, nonparaventricular recurrence.

As shown in **Figure 2** and **Table 6**, there were more patients with strong positive expression of S-100 in the local recurrence group, and the difference was statistically significant (76.19% vs. 52.27%, χ^2^ = 5.876, p=0.015). The occurrence of positive and strong positive expression of PCNA in the nonlocal recurrence group was significantly higher than that in the local recurrence group (60.71% vs. 33.33%, χ^2^ = 4.364, p=0.037). In comparing positive and strong positive P53 expression with weak positive P53 expression, there was a significant difference between the groups (84.62% vs. 50.00%, χ^2^ = 6.872, p=0.009). The expression of Ki-67 in the nonlocal recurrence group was significantly higher than that in the local recurrence group (20 (10-40) vs. 10 (5-30), U=2.436, p=0.015). Moreover, there was no significant difference in the expression of GFAP, Olig-2, Syn, NSE, Pgp170, Vimentin, MGMT, MMP-9, IDH-1, EGFR, GST-π, and TOPO II between the local recurrence group and the nonlocal recurrence group. The same analysis was further done separately for WHO II/III or WHO IV patients respectively, however, no molecular markers showed significantly different expression lever between the local and nonlocal recurrence cases for these two subgroups (data not shown).

**Table 6:** Statistically significant molecular markers between the groups.

	S-100		PCNA		P53		MGMT	
	LR	NLR	LR	NLR	LR	NLR	PR	NPR
Negative (–)		1	6	3	15	17	12	9
weak positive (+-)		3	14	8	12	4	14	1
Positive (+)	10	18	7	11	7	10	4	
strongly positive (++);	32	23	3	6	5	12		
total	42	44	30	28	39	43	30	10
χ2-value	5.876		4.364		6.872#		5.564	
p-value	0.015*		0.037*		0.009**		0.017*	

*p < 0.05, **p < 0.01, #weak positive vs. positive& strongly positive; LR, Local recurrence; NLR, non-local recurrence; PR, Paraventricular recurrence; LPR, non-paraventricular recurrence.

The occurrence of positive and weak positive expression of MGMT was significantly higher in the paraventricular recurrence group than in the nonparaventricular recurrence group (60% vs. 10%, U=5.564, p=0.017). In addition, there was no significant difference in the expression of Ki-67, S-100, PCNA, P53, GFAP, Olig-2, Syn, NSE, Pgp170, Vimentin, MMP-9, IDH-1, EGFR, GST-π, and TOPO II between the paraventricular recurrence group and the nonparaventricular recurrence group. Additionally, no molecular markers showed significantly different expression lever between the paraventricular and non paraventricular recurrence group either for the WHO II/III or WHO IV patients respectively (data not shown).

According to the final model of multivariate Cox regression analysis, which take the development of different patterns of recurrence as the outcome measures: hazard ratio (HR) for high expression level of PCNA associated with non-local recurrence was 3.43 (95% CI, 1.15, 10.24), and HR for high expression level of MGMT associated with paraventricular recurrence was 2.64 (95% CI, 1.15,6.08) (Seen in **Table 7**).

**Table 7:** Independent predictive factors for different recurrence groups.

		Univariate analysis					Multivariate analysis				
		B	HR	95%CI		P	B	HR	95%CI		P
NLR vs LR											
	Ki67	0.021	1.02	1.00	1.04	0.033*	0.008	1.01	0.98	1.04	0.572
	Age	0.854	2.35	0.98	5.66	0.057	0.866	2.38	0.69	8.24	0.172
	S100	-1.072	0.34	0.14	0.86	0.023*	-0.851	0.43	0.12	1.50	0.184
	PCNA	1.128	3.09	1.06	9.04	0.039*	1.234	3.43	1.15	10.24	0.027*
PR vs NPR											
	RTV	0.015	1.02	1.00	1.03	0.083	0.003	1.00	1.00	1.01	0.119
	WHO grades	1.620	5.06	1.24	20.63	0.024*	0.036	1.04	0.34	3.12	0.949
	MGMT	2.603	13.50	1.51	120.78	0.02*	0.971	2.64	1.15	6.08	0.022*

*p < 0.05; LR, Local recurrence; NLR, non-local recurrence; PR, Paraventricular recurrence; NPR, non-paraventricular recurrence; HR, hazard ratio; RTV, Recurrent tumor volume.

## Discussion

In this study, we identified significant correlations between clinical characteristics and molecular markers and glioma recurrence location. Previous studies revealed the mechanisms of glioma recurrence mostly regarding cellular pathways, and clinical studies showed that different recurrence patterns had different prognosis. Moreover, our analysis shows that age, postoperative treatment, and Ki-67, P53, PCNA, and S-100 expression are associated with local recurrence, and WHO grade, tumor size, and MGMT expression are associated with paraventricular recurrence. These results complement those of previous studies of molecular mechanisms and hopefully assist in predicting, diagnosing, and treating recurrent glioma.

Our results show that patients aged >40 years tended to experience nonlocal recurrence; in the present study, older patients may have a worse prognosis, which is consistent with the high degree of malignancy of nonlocal recurrence (12, 13). Previous studies showed that patients’ WHO grades of marginal/local recurrence were in accordance with distance/multirecurrence (12, 14), which is roughly similar to our results. However, sex and time to recurrence did not differ according to local recurrence or paraventricular recurrence. In the paraventricular recurrence group, the number of WHO grade IV patients and the tumor volume were greater than those in the nonparaventricular recurrence group, which explains why the prognosis of paraventricular glioma patients is worse (15). It has been reported that ventricular or periventricular high grade gliomas (HGG) always represent unique clinical features, for it involve the ventricular zone or subventricular zone (V-SVZ) (16). V-SVZ is known to harbor neural stem cells of a multipotent nature, which increases the propensity to generate aggressively proliferating tumors (16). Thus, the ventricular or periventricular HGG always represented more aggressive proliferating features, more resistance to the therapy and poor prognosis (17). By our analysis, it can be found that the volume of recurrent tumors in paraventricular recurrences was seven times bigger than non-paraventricular recurrences, the possible explanation may be that if recurrence occurred, the recurrent tumor in periventricular zone proliferated more aggressively and represented more resistance to therapy than that in non-paraventricular zone as well, which could also be attributed to the special feature of V-SVZ, although the detailed mechanism for the recurrence was still unclear.

In the present study, postoperative treatment was significantly associated with the glioma recurrence location. When patients receive combined radiotherapy and chemotherapy, they tend to experience nonlocal recurrence. It seems that standard treatment cannot benefit patients because this experiment lacks a control group consisting of nonrecurrent patients. This result may be because the range of radiotherapy is limited to the primary glioma location. Moreover, compared with chemotherapy only, the combination of radiotherapy and chemotherapy is more effective in suppressing local tumor growth, mainly due to the better in-field (as for the radiotherapy) therapeutic effect for the local focus, thus more nonlocal recurrence occurred for these group of patients. Additionally, We speculate that standard postoperative treatment changes the glioma environment of the primary tumor location, which leads to the genetic alteration of residual glioma cells, and then patients experience nonlocal recurrence of glioma (18). However, this hypothesis and its molecular mechanism require further experiments. The significance of this result is finding one kind of glioma that could resist standard postoperative treatment and recur in other locations, which reflects the high diversity of glioma. In addition, in our study, the extent of surgery did not influence the recurrence pattern, while in previous research, STR could cause local recurrence (19); however, this discrepancy may have resulted from our sample size being too small. In addition, although the expression of molecular markers significantly differed between the local recurrence group and the nonlocal recurrence group, however, the clinical course between the two groups was similar. These results may be explained by the limitation of the small sample-size and the follow-up method by fixed intervals as well.

Ki-67, which was initially detected as an autoantibody in the blood of leukemia patients in 1992, is expressed during the G1, S, G2, and M stages of the cell cycle. Ki-67 can be detected in cells with active proliferation and is a marker that measures tumor proliferation (20). Present clinical research has reported that a high Ki-67 proliferation index is related to glioma recurrence after initial surgery (21). Moreover, some studies found a correlation between Ki-67 expression and WHO grades, thus playing a role in predicting the prognosis of patients (22). In our results, the expression of Ki-67 in the nonlocal recurrence group was significantly higher than that in the local recurrence group. Ki-67, which reflects the proliferation capacity of tumor cells, may be an important basis of glioma distant recurrence.

PCNA, which is an antigen characteristic of proliferating cells that is expressed in cell nuclei during the S phase of the cell cycle, is a widely recognized cell proliferation marker that also serves as a prognostic indicator for a variety of tumors, including glioma (23). Previous clinical research reported the high expression of PCNA in metastatic glioma, which is consistent with our results. In an animal experiment, the GDNF stimulation of rat glioma cells increased the expression of PCNA and Ki-67 and enhanced the proliferation of tumor cells, and in our research, the expression of both PCNA and Ki-67 were increased in patients with nonlocal recurrence (24). A previous *in vitro* study showed that the PCNA-MGMT complex forms in glioma cells and could be inhibited by p21, a cell cycle inhibitor (25). Moreover, downregulating SCD1 could decrease the expression of PCNA and MMP-9; thus, the proliferation and invasion of glioma cells were weakened (26). However, in our results, MGMT and MMP-9 did not increase in patients with nonlocal recurrence; we infer that these two mechanisms are not related to the nonlocal recurrence of glioma.

S-100 and GFAP are structural cytoskeleton proteins expressed by astroglial and neuronal stem cells (27). S-100, which is an acidic calcium-binding protein that is mainly secreted by Schwann cells to the peripheral nervous system, has trophic effects on glial cells and neurons (28). Researchers have found that S100B promotes glioma growth *via* tumor-associated macrophages (29) and that the suppression of S100B inhibits glioma growth (30). In our research, the high expression of S-100 was related to local recurrence, which indicates that S-100 may be an important molecular marker for predicting recurrence patterns. At present, we cannot explain the reason for this result, and more studies are needed to explore the correlation between S-100 expression and the tumor recurrence pattern.

P53 is one of the most well-known tumor suppressor proteins to date and has been implicated in almost every cancer (31). Our results indicated that the expression of P53 in glioma patients with nonlocal recurrence is higher than that in glioma patients with local recurrence. Many studies have shown that changes in P53 cell pathways could increase the invasion and metastasis of glioma cells (32), which is consistent with our research findings. In addition, some studies have reported that standard postoperative treatment could change the P53 signaling pathway, which is deemed to cause glioma recurrence (18). However, in some studies, the activation of P53 could be a therapeutic strategy for GBM (33). Present studies of glioma are conflicting because the P53 pathway functions in many different cellular responses, such as cell cycle regulation, apoptosis, differentiation, and DNA damage response, and the prognostic and predictive response of this protein in glioma recurrence is still largely undetermined (34).

By Cox regression model, univariate regression analysis also showed that PCNA, S100 and Ki67 predict the non-local recurrence (NLR) pattern, which inferred that the NLR was attributed to increased proliferation and invasion, which can be indexed by these molecular markers. The reason why multivariate Cox regression only detected the expression of PCNA predicted the nonlocal recurrence may be due to the inherent correlation between S100/Ki67 and the PCNA. It has been found that the overexpression of p53-protein may correlate with the expression of PCNA or Ki-67, which were established as the markers of cell proliferation, in human astrocytomas (35). Expression of S100 and PCNA were both indicated to be significantly differed in astrocytic tumors of different types and grades (36). Therefore, the univariate and the multivariate Cox regression results, together suggested that the increased proliferation and invasion was the main reason for the nonlocal recurrence of the gliomas.

Our results showed that the expression of MGMT between the defined groups was significantly different, and the chi-square test demonstrated that the high expression of MGMT may be correlated with paraventricular recurrence. In addition, both the univariate and multivariate Cox regression analysis further indicated the high expression level of MGMT to be the significant predictor for the paraventricular recurrence. However, only univariate Cox regression analysis found WHO grades predict the paraventricular recurrence, while univariate analysis did not. Actually, though WHO grade may be correlated to the prognosis for the patients, it can not predict the therapeutic response for individual patient. Patients with unmethylated MGMT have a short overall survival because of its correlation with TMZ resistance (37), thus our results indicated that the therapeutic resistance, but not the shorted survival/earlier recurrence simply, may be the key factor inducing the periventricular recurrence. Current radiographic research indicated that most GBMs grow into the periventricular white matter regions adjacent to the subventricular zone (SVZ) (38). Basic research has indicated that the neural stem cells of the SVZ are correlated with glioma recurrence (39). In addition, in a clinical study, paraventricular or ventricular glioma was also associated with short overall survival (15). Our research indicated the difference in the paraventricular recurrence of glioma in terms of molecular markers, suggesting the tumors with more resistance to the TMZ chemotherapy more likely tend to develop the paraventricular recurrence, while the detailed mechanism need to be further probed. It is a resistance mechanism of TMZ in MGMT-positive patients that causes paraventricular recurrence, and previous research reported that the microenvironment of TMZ resistance is related to the migration of glioma cells (40, 41). Other potential MGMT-related mechanisms may directly lead to paraventricular recurrence, which we cannot ensure at present. Paraventricular glioma is difficult to operate on or diagnose, and endoscopy is the most common method for biopsy, which is difficult and not suitable for most patients (42). We researched clinical characteristics and molecular markers in recurrent paraventricular glioma, which provide guidance for diagnosis and therapy. The expression of IDH-1 between the groups was significantly different (p=0.069), but this result may require a larger sample size for confirmation.

On the other hand, we can not find difference of expression for many molecular markers between divergent recurrence groups. As for the makers such as GFAP, Olig-2, and NSE, the negative findings indicated that different recurrence patterns do not result from the cell types from which the tumor origins. Besides, though MMP-9, EGFR, GST-π, and TOPO II all play crucial roles in the progression, invasion, migration and dissemination of gliomas, their expression levels do not differed among divergent recurrence groups, indicating that the recurrence pattern may not be determined by these molecules. IDH-1, which predicts the prognosis of the gliomas, however, seemed to have no relations with the recurrence modes of the tumors.

### Limitation

This is a retrospective single-center study, which provides weaker evidence than prospective randomized controlled trials. Multicenter and large sample studies are needed. Specifically, the relatively sample size limit the further analysis when divided the patients into subgroups, e.g., WHO grade IV vs. II/III cases. In addition, we detected only the expression of MGMT rather than MGMT promoter methylation status. Methylation of MGMT gene promoter decreases the normal DNA-repair function of the MGMT enzyme, which can make tumors more susceptible to radiation or alkylating agent-based therapy. Thus to be exact, it is the MGMT gene promoter methylation status which directly predicts the therapeutic response of the tumors. The MGMT expression level detected by the present study was just correlated to but not equivalent to the MGMT gene promoter methylation status, which would affect the reliability of our results. Thirdly, the recurrence time-points identified by the image follow-up with intervals for several months, may be not exact or precise enough, which may influence the Cox regression analysis results. Finally, many various and divergent small groups further decrease the sample size for different recurrence pattern, which may reduce the power of statistical analysis.

## Conclusion

In summary, our data indicate that Ki-67, S-100, PCNA, and P53 expression levels in glioma are associated with local/nonlocal recurrence, and the MGMT expression level is associated with paraventricular/nonparaventricular recurrence. It can be speculated that the molecular markers represent potential clinical markers for nonlocal recurrence or paraventricular recurrence. In addition, we found that age, postoperative treatment, recurrent tumor size, and WHO grade were significantly correlated with the defined groups. These results may be helpful for assisting in the prediction of glioma recurrence patterns

## Data Availability Statement

The raw data supporting the conclusions of this article will be made available by the authors, without undue reservation.

## Ethics Statement

The studies involving human participants were reviewed and approved by Tang Du Hospital of Fourth Military Medical University institutional review board. Written informed consent to participate in this study was provided by the participants’ legal guardian/next of kin.

## Author Contributions

LW, YQ, SG, YS, and GZ contributed with study design, data analysis, interpretation of findings, and writing of the manuscript. YC, SL, JL, WG, and LG contributed with data collection. PJ, ML, FF, YW, and YZ contributed with literature research. All authors contributed to the article and approved the submitted version.

## Conflict of Interest

The authors declare that the research was conducted in the absence of any commercial or financial relationships that could be construed as a potential conflict of interest.
