# Supplementary material for: Molecular Pathological Markers Correlated With the Recurrence Patterns of Glioma
Source: Front Oncol. 2021 Jan 8;10:565045. doi: 10.3389/fonc.2020.565045 (PMC7873968; doi:10.3389/fonc.2020.565045)
Supplement: Supplementary file 1 [file DataSheet_1.docx]

Supplementary table 1: comparison of clinical factors in WHOII/III patents with different recurrence patterns

|  | Local recurrence n=10 | Nonlocal recurrence n=13 | χ^2^- or U- value | p-value | Paraventricular recurrence n=6 | Nonparaventricular recurrence n=7 | χ^2^- or U- value | p-value |
| --- | --- | --- | --- | --- | --- | --- | --- | --- |
| Sex |  |  |  |  |  |  |  |  |
| male | 4 | 5 | 0.06 | 0.940 | 2 | 7 | 6.741 | 0.021 |
| female | 6 | 8 |  |  | 4 | 0 |  |  |
| Age |  |  |  |  |  |  |  |  |
| median | 34(28,45) | 44(35.5,53) | 1.537 | 0.124 | 48(38.75,57.25) | 43(26,54) | 0.474 | 0.534 |
| <40 | 8 | 4 | 5.490 | 0.019* | 1 | 3 | 1.04 | 0.308 |
| ≥40 | 2 | 9 |  |  | 5 | 4 |  |  |
| Post-operative treatment |  |  |  |  |  |  |  |  |
| Observe | 3 | 5 | 2.637 | 0.267 | 2 | 3 | 0.124 | 0.94 |
| Chemotherapy only or radiotherapy only | 5 | 2 |  |  | 1 | 1 |  |  |
| Combine radiotherapy and chemotherapy | 2 | 6 |  |  | 3 | 3 |  |  |
| Extent of surgery |  |  |  |  |  |  |  |  |
| Gross-total resection | 4 | 5 | 0.06 | 0.940 | 1 | 4 | 2.136 | 0.135 |
| Subtotal resection | 6 | 8 |  |  | 5 | 3 |  |  |
| Recurrent tumor volume(mm^3^) | 38.05(6.99,94.5) | 34.43(10.80,111.69) | 0.377 | 0.706 | 94.63(34.14,143.65) | 12.04(1.35,70.00) | 2 | 0.046* |
| Recurrent tumor location |  |  |  |  |  |  |  |  |
| frontal | 5 | 5 | 2.642 | 0.709 | 5 | 3 | 6.281 | 0.128 |
| temporal | 3 | 3 |  |  | 0 | 2 |  |  |
| parietal | 0 | 2 |  |  | 4 | 0 |  |  |
| occpital | 1 | 2 |  |  | 1 | 1 |  |  |
| other | 2 | 6 |  |  | 5 | 1 |  |  |
| Time to recurrence (months) | 10(7,25) | 6(4.5,12) | 1.858 | 0.063 | 9(4.5,16) | 5(4,8) | 0.387 | 0.445 |

P<0.05.

Supplementary table 2: comparison of clinical factors in WHOⅣ patents with different recurrence patterns

|  | Local recurrence n=33 | Nonlocal recurrence n=32 | χ^2^- or U- value | p-value | Paraventricular recurrence n=26 | Nonparaventricular recurrence n=6 | χ^2^- or U- value | p-value |
| --- | --- | --- | --- | --- | --- | --- | --- | --- |
| Sex |  |  |  |  |  |  |  |  |
| male | 16 | 18 | 0.251 | 0.616 | 13 | 1 | 2.201 | 0.138 |
| female | 16 | 14 |  |  | 13 | 5 |  |  |
| Age |  |  |  |  |  |  |  |  |
| median | 43(32.25,53) | 48(38.25,54) | 1.095 | 0.273 | 49.5(38.5,54.25) | 42(36.25,48.25) | 0.277 | 0.285 |
| <40 | 13 | 9 | 1.108 | 0.292 | 6 | 3 | 1.748 | 0.186 |
| ≥40 | 19 | 23 |  |  | 20 | 3 |  |  |
| Post-operative treatment |  |  |  |  |  |  |  |  |
| Observe | 7 | 6 | 3.458 | 0.178 | 5 | 1 | 1.99 | 0.394 |
| Chemotherapy only or radiotherapy only | 13 | 7 |  |  | 7 | 0 |  |  |
| Combine radiotherapy and chemotherapy | 12 | 19 |  |  | 14 | 5 |  |  |
| Extent of surgery |  |  |  |  |  |  |  |  |
| Gross-total resection | 19 | 13 | 2.25 | 0.134 | 11 | 2 | 0.163 | 0.687 |
| Subtotal resection | 13 | 19 |  |  | 15 | 4 |  |  |
| Recurrent tumor volume(mm^3^) | 26.87(10.53,79.20) | 36.62(8.05,79.68) | 0.155 | 0.877 | 48.90(11.14,93.78) | 8.44(2.86,59.30) | 0.097 | 0.104 |
| Recurrent tumor location |  |  |  |  |  |  |  |  |
| frontal | 14 | 19 | 1.585 | 0.931 | 13 | 3 | 2.519 | 0.698 |
| temporal | 8 | 7 |  |  | 7 | 1 |  |  |
| parietal | 3 | 4 |  |  | 2 | 0 |  |  |
| occpital | 0 | 1 |  |  | 1 | 0 |  |  |
| other | 7 | 11 |  |  | 7 | 4 |  |  |
| Time to recurrence (months) | 9(7,17.75) | 10.5(5,18.75) | 0.061 | 0.952 | 9(4.75,16) | 18.5(6,26.5) | 0.256 | 0.264 |
